# Supplementary material for: Two-component energy spectrum of cuprates in the pseudogap phase and its evolution with temperature and at charge ordering
Source: Sci Rep. 2015 Feb 17;5:8524. doi: 10.1038/srep08524 (PMC4330546; doi:10.1038/srep08524)
Supplement: Supplementary Information [file srep08524-s1.pdf]

Supplementary information for "Two-component energy spectrum of cuprates in the pseudogap phase and its evolution with temperature and at charge ordering"

Lev P. Gor'kov and Gregory B. Teitel'baum

Table of contents

SI1 The T^2 - resistivity regime

SI2 Model of Fermi arcs and summary of theoretical results

SI3 The notion of two components in the effective number of Hall carriers.

SI1 The T^2 - resistivity regime

In the Drude formula $\rho(T)=[m*/ne^2\tau(T)]$ the FL-like T^2 contribution for layered materials $\Delta\rho(T)$ can be presented in the form: $(s/c)(e^2/\pi\hbar)\Delta\rho(T)=const\times\left(T/\varepsilon_F\right)^2$.

(c is the lattice constant perpendicular to the CuO_2-plane in cuprates; s -is the number of conducting layers in the unit cell). For LSCO [22] use of the Drude formula gave for the energy Fermi the meaninglessly small value $\varepsilon_F\sim 150\div 200K$. Same inconsistency was found in [16] for a broader class of UD cuprates. For instance, from the resistivity data in Fig. 3 [16], say, for YBCO one finds $\varepsilon_F\approx(const)^{1/2}290K$, while $T^{**}=190$ K . As emphasized before [22], in theory, the T^2 - dependence in resistivity can signify a FL regime *only* as long as $T<<\varepsilon_F$ in the *whole* temperature interval. That is, the Fermi energy *must be larger than temperature in whole interval*.

SI2 Model of Fermi arcs and summary of theoretical results [23]

Inside the Fermi arc the energy spectrum of carriers is:

$$\varepsilon(\vec{p})=\pm v_F(p^{\parallel}\mp p_F)-(p^{\perp})^2/2m \quad (1).$$

The arc's length is $\Delta\varphi p_F$; p_F, v_F are the Fermi momentum and the Fermi velocity, respectively; $p^{\parallel}, p^{\perp}$ stand for the parallel and the perpendicular momentum projections on direction of one of the two diagonals; the Γ -point lies in the area of the BZ occupied by electrons; the choice of sign ($\pm$) for each Fermi arc in Fig.1a(insert) is self-evident.

Resistivity and the Hall coefficient are found by solving the kinetic equation

$$e(\vec{E}+\frac{1}{c}[\vec{v}\times\vec{H}])\vec{\nabla}_{\vec{p}}n(\vec{p})=(\frac{dn}{dt})_{coll}\ . \quad (2)$$

The textbook integral $(dn/dt)_{coll}$ balances "gains" and "losses" at the *e-e* collisions:

$$(\frac{dn_{\vec{p}}}{dt})_{coll}=2\iint|V(\vec{p}_1\vec{p}_3;\vec{p}\vec{p}_2)|^2\frac{d^2\vec{p}_1d^2\vec{p}_2}{(2\pi)^4}\delta(\varepsilon_1+\varepsilon_3-\varepsilon-\varepsilon_2)\{(gains)-(losses)\} \quad (3)$$

where $\varepsilon_i\equiv\varepsilon_i(\vec{p}_i)$; $"gains"=n(\vec{p}_1)n(\vec{p}_3)[1-n(\vec{p})][1-n(\vec{p}_2)]$ and the obvious permutations give the expression for $"losses"$.

At small Fermi arcs $\Delta\varphi<<1$ (confirmed experimentally) the kinetic equation was solved analytically. The tetragonal symmetry simplifies the pattern of currents in the model: when the electric field is along one of the diagonals of the BZ the Hall currents flow along the second diagonal. Processes with the Umklapp scattering of the two carriers are shown by arrows in insert in Fig.1a.

With the energy spectrum (1) the conservation of momentum along the spatial diagonals $p^{||}+p_2^{||}-p_1^{||}-p_3^{||}-K^{||}=0$ can be presented through the energy variables as

$$p^{||}+p_2^{||}-p_1^{||}-p_3^{||}-K^{||}\equiv v_F^{-1}[\varepsilon(\vec{p})+\varepsilon(\vec{p}_2)+\varepsilon(\vec{p}_1)+\varepsilon(\vec{p}_3)-\Sigma]=0 \quad (4)$$

where Σ is the quadratic function of the perpendicular components of the momentum only

$$\Sigma\equiv\{(p^{\perp})^2+(p_2^{\perp})^2+(p_1^{\perp})^2+(p_3^{\perp})^2\}/2m^*-4\varepsilon_F[K/p_F-4] \quad (5)$$

(In the above $\vec{K}$ is the Umklapp vector along direction of one of the diagonals in the BZ). In turn, at the integrations over the momentum $\vec{p}_i$ one can use the relation [23]:

$$\frac{d^2\vec{p}}{(2\pi)^2}\Rightarrow\frac{d\varepsilon dp^{\perp}}{v_F(2\pi)^2} \quad (6)$$

The results can be reduced to the familiar form introducing the drift velocity $\vec{u}$:

$$\frac{1}{\tau}\vec{u}=\frac{(-|e|)}{m^*}(\vec{E}+\frac{1}{c}[\vec{u}\times\vec{H}]) \quad (7)$$

The inverse mean free time $1/\tau=1/\tau_{FA}+1/\tau_{def}$ is sum of the contribution due to interactions between holes $1/\tau_{FA}$ and scattering of holes on defects $1/\tau_{def}$. Accordingly, the mobility is $\mu=e\tau/m^*$.

In the clean limit the carriers (holes) on the Fermi arcs interact via the short-range (screened) Coulomb interactions. From [23]:

$$\tau_{FA}(T) = \frac{3\hbar\Delta\varphi\varepsilon_F}{8\pi |V(1;2)|^2 T^2 \sqrt{2[(K/4p_F)-1]}} . \qquad (8)$$

For the in-layer resistance

$$\rho_{2D}(T) = \left(\frac{\pi\hbar}{e^2}\right) \frac{4\pi^2 |V(1;2)|^2 \sqrt{2[(K/4p_F)-1]}}{3(\Delta\varphi)^2} \left(\frac{T}{\varepsilon_F}\right)^2 . \qquad (9)$$

The expression is proportional to square of the renormalized matrix elements for inter-electronic collisions $V(1;2)$; $m* = Z^{-1}m$ is the renormalized band mass and Z is the residue factor for the Green function of excitations. The combination $\varepsilon_F = p_F^2 / 2m^*$ is the renormalized Fermi energy at the nodal point (see [23]).

For the Hall coefficient one finds:

$$R_H = \frac{1}{|e| c n_{eff}} > 0 , \quad (10)$$

where for the isotropic energy spectrum $n_{eff} = \frac{\Delta\varphi p_F^2}{\pi^2} (\frac{s}{c})$. Formally, expression (10) for R_H is applicable only in the limit of a weak magnetic field $\omega_c^* \tau << 1$. Here $\omega_c^* = (eH / m^* c)$ and $m*$ is the *effective* mass for *holes on the Fermi arcs*. Later, the arguments were given that for the Fermi arcs the expression (10) for the Hall coefficient remains correct in the opposite limit of *strong* magnetic fields $\omega_c^* \tau >> 1$.

For scattering on the static defects one finds:

$$\frac{1}{\tau_{def}} = \frac{N_{def} |W|^2}{v_F \pi^2} . \quad (11)$$

In (11) $|W|^2$ is square of the matrix element for scattering on the impurity potential and N_{def} is density of the defects.

Making use of Eq. (7) one finds for the transverse magneto-resistance the expression

$$\frac{\delta\rho}{\rho_0} = \frac{\rho(H) - \rho_0}{\rho_0} = (\tau_{FA}\omega_c^*)^2 . \quad (12)$$

For the two-component system of holes at FAs and electrons on the pocket we had to solve system of the two kinetic equations. The pocket is assumed to be at the Γ-point of the BZ. Due to smallness of its size the Umklapp processes are irrelevant and in the corresponding collision integral are kept only the terms for scattering of electrons on the Fermi arcs:

$$(dN_2 / dt) = 2\sum_{1,3,4} | I(p_2, p_1; p_3, p_4)|^2 \{-N_2 n_1(1-n_3)(1-N_4) + (1-n_1)(1-N_2)n_3 N_4\}$$
$$\times\delta(\varepsilon_1 + E_2 - \varepsilon_3 - E_4)\delta(\vec{p}_1 + \vec{P}_2 - \vec{p}_3 - \vec{P}_4) \qquad (13)$$

(To save on notations, the upper and low cases are used to distinguish between electrons and holes, correspondingly).

After substituting $\delta N_i(\vec{p}_i) = (\vec{p}_i \cdot \vec{v}_e)(\frac{1}{4T})ch^{-2}(\frac{E_i}{2T})$ in (13) and integrating both the left and the right sides of the two kinetic equations over all momentums, one arrives to the following system of equations for the *drift velocities* $\vec{u}$ and $\vec{v}_e$ of holes on FAs and electrons, respectively:

$$\frac{1}{\tau_h}\vec{u} = \frac{-|e|}{m^*}\left(\vec{E} + \frac{1}{c}[\vec{u}\times\vec{H}]\right) \qquad (14a)$$

$$\frac{1}{\tau_{e,i}}\vec{v}_e + \frac{1}{\tau_{eh}}(\vec{v}_e - \vec{u}) = \frac{|e|}{m^*}\left(\vec{E} + \frac{1}{c}[\vec{v}_e\times\vec{H}]\right) \qquad (14b)$$

Here Eq. (14a) is the same Eq. (7) with $1/\tau_h \equiv 1/\tau = 1/\tau_{FA} + 1/\tau_{def}$; $1/\tau_{eh}$ stands for the parameter coupling the two sub-systems and is most essential in the clean limit. Derivation of the explicit expression for $1/\tau_{eh}$ from Eq. (13) turned out a trying process. We were able to find its expression with the accuracy up to a numerical factor only. For the final results it is essential that by the order of magnitude $1/\tau_{eh}$ and $1/\tau_{FA}$ are of the same order and both proportional to T^2.

Eqs. (14a, b) allow calculating the conductivity and the Hall coefficient. In the main text the results are discussed in the limit $n_e << n_{eff}$ (Eqs. (2), (3) and below).

SI3 The notion of two components in the effective number of Hall carriers.

As it has been demonstrated in [27, 28] the analysis of the data [20] on the Hall coefficient, $R_{Hall}(T, x)$, for $La_{2-x}Sr_xCuO_4$ leads to the notion of two components in the effective number of charge carriers n_{Hall} which may be presented as $n_{Hall} = n_0(x) + n_1 \exp[-\Delta(x)/T]$. The first component is temperature independent, while the second one bears the activation character. Shown in Fig. **S**1 are the fits of this equation to $R_{Hall}(T, x)$ temperature dependences [20] together with the fitting parameters values for few Sr concentrations *x*.

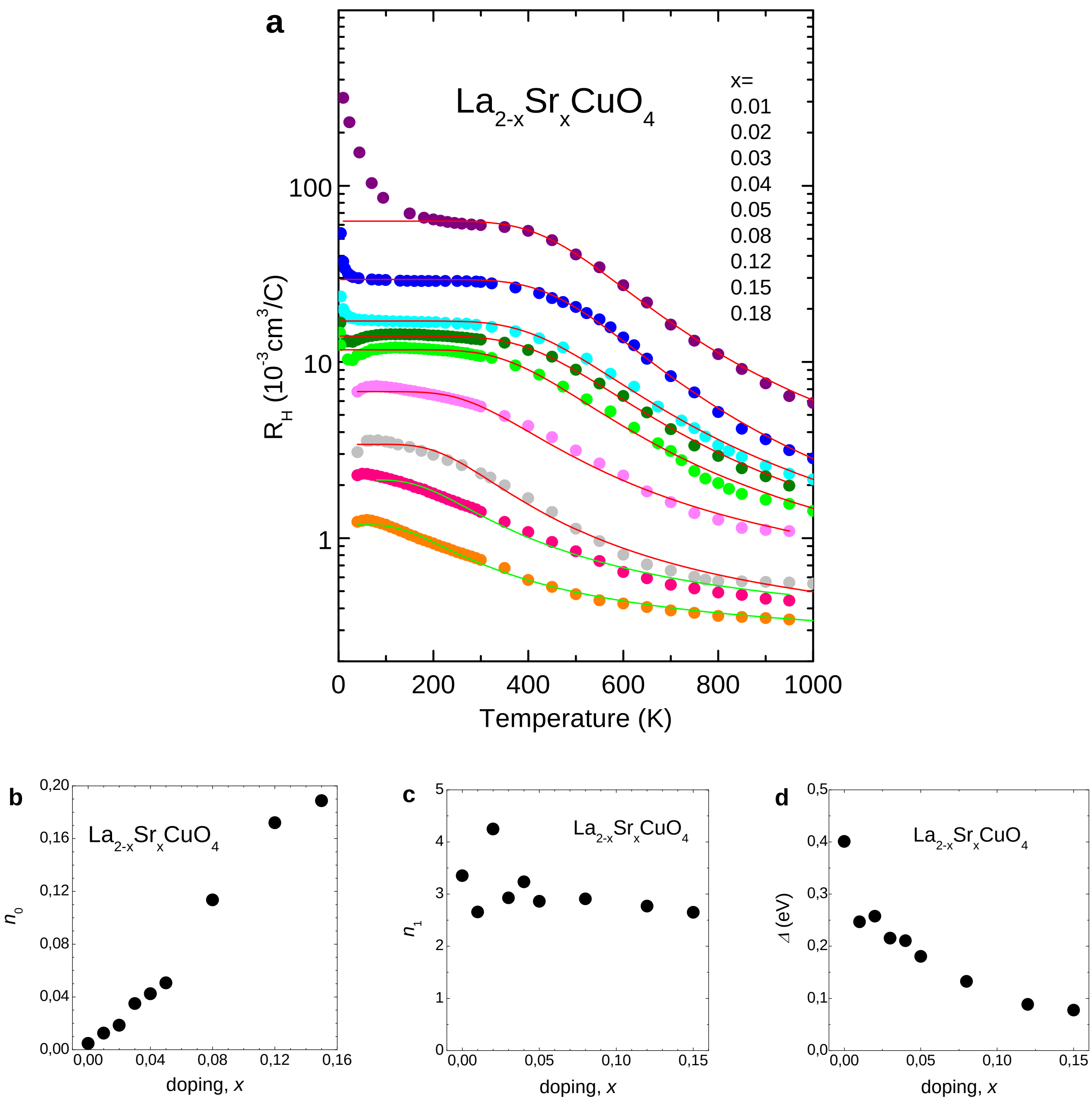

Figure S1. The notion of two components in the effective number of Hall carriers. a. The R_H temperature dependence [20] at different doping x. The continuous lines are the fits to Eq. (1); **b.** The doping dependence of the temperature independent term $n_0(x)$; **c.** The doping dependence of the coefficient $n_1(x)$ in the activation contribution; **d.** The doping dependence of the activation energy $\Delta(x)$.
